# Supplementary material for: Effectiveness and Safety of Ayurvedic Medicines in Type 2 Diabetes Mellitus Management: A Systematic Review and Meta-Analysis
Source: Front Pharmacol. 2022 Jun 8;13:821810. doi: 10.3389/fphar.2022.821810 (PMC9213670; doi:10.3389/fphar.2022.821810)
Supplement: Supplementary file 1 [file DataSheet1.zip › Supplementary Material/Forest and Funnel Plots/Portulaca oleracea/TC.pdf]

Portulaca oleracea - TC

Study	Intervention N	Intervention Mean	Intervention SD	Comparator N	Comparator Mean	Comparator SD	Mean Diff. with 95% CI	Weight (%)
Farzanegi 2014	14	161.15	25.07	14	180.00	32.30	-18.85 [-40.27, 2.57]	37.79
Dehghan 2016	16	163.75	49.73	16	189.45	52.37	-25.70 [-61.09, 9.69]	13.84
Wainstein 2016	31	189.20	36.30	32	205.90	40.20	-16.70 [-35.63, 2.23]	48.36
**Overall**							-18.76 [-31.93, -5.59]	

Heterogeneity: $\tau^2 = 0.00$, $I^2 = 0.00\%$, $H^2 = 1.00$

Test of $\theta_i = \theta_j$: Q(2) = 0.19, p = 0.91

Test of $\theta = 0$: z = -2.79, p = 0.01

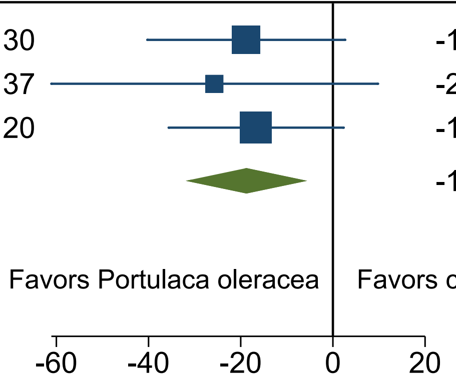

Random-effects REML model
